# Seroconversion rate and socio-economic and ethnic risk factors for SARS-CoV-2 infection in children in a population-based cohort, Germany, June 2020 to February 2021

**DOI:** 10.2807/1560-7917.ES.2022.27.37.2101028

**Published:** 2022-09-15

**Authors:** Folke Brinkmann, Hans H Diebner, Chantal Matenar, Anne Schlegtendal, Lynn Eitner, Nina Timmesfeld, Christoph Maier, Thomas Lücke

**Affiliations:** 1 University Children's Hospital, Ruhr University Bochum, Bochum, Germany; 2 Department of Medical Informatics, Biometry and Epidemiology, Ruhr University Bochum, Bochum, Germany

**Keywords:** SARS- CoV-2, seroconversion, children, migration, socioeconomic

## Abstract

**Introduction:**

Socio-economic and ethnic background have been discussed as possible risk factors for SARS-CoV-2 infections in children. Improved knowledge could lead to tailored prevention strategies and help improve infection control.

**Aim:**

We aimed to identify risk factors for SARS-CoV-2 infections in children in the first and second wave of the pandemic.

**Methods:**

We performed an observational population-based cohort study in children (6 months–18 years) scheduled for legally required preventive examination and their parents in a metropolitan region in Germany. Primary endpoint was the SARS-CoV-2 seroconversion rate during the study period. Risk factors assessed included age, pre-existing medical conditions, socio-economic factors and ethnicity.

**Results:**

We included 2,124 children and their parents. Seroconversion rates among children in all age groups increased 3–4-fold from June 2020 to February 2021. Only 24 of 58 (41%) seropositive children reported symptoms. In 51% of infected children, at least one parent was also SARS-CoV-2-positive. Low level of parental education (OR = 3.13; 95% CI: 0.72–13.69) non-significantly increased the risk of infection. Of the total cohort, 38.5% had a migration background, 9% of Turkish and 5% of Middle Eastern origin, and had the highest risk for SARS-CoV-2 infections (OR = 6.24; 95% CI: 1.38–28.12 and OR = 6.44 (95% CI: 1.14–36.45) after adjustment for other risk factors.

**Conclusion:**

In the second half of 2020, seroprevalence for SARS-CoV-2 in children increased especially in families with lower-socioeconomic status. Culture-sensitive approaches are essential to limit transmission and could serve as a blueprint for vaccination strategies.

**Public Health impact of this article**

**What did you want to address in this study?**

This study was initiated to estimate seropositivity for SARS-CoV-2 during the first year of the pandemic in an unselected cohort of children and adolescents and identify risk factors for infection.

**What have we learnt from this study?**

Neither age nor sex were risk factors for SARS-CoV-2 infection, whereas contact to infected family members and, independent of other findings, a Middle Eastern or Turkish migration background increased the risk for infection.

**What are the implications of your findings for public health?**

Populations at risk for infection should be the focus of public health interventions to improve infection control and other preventative measures (e.g. vaccine uptake).

## Introduction

The rate of infections with severe acute respiratory syndrome coronavirus 2 (SARS-CoV-2) in children was discussed controversially during the first months of the pandemic. Children are frequently oligo- or asymptomatic and therefore undertested and underdiagnosed. Seropositivity studies, however, reveal that the rate of SARS-CoV-2 infections in adults and children is similar [1-3].

Most transmissions probably take place in households, where contacts are most intense [2,4]. Crowded living conditions and poverty have been associated with a higher rate of SARS-CoV-2 infection both in adults and children as well as increased morbidity and mortality in Europe and in the United States (US) [5,6]. Ethnic origin has also been described as a risk factor for hospitalisation for coronavirus disease (COVID-19) in children in the US [7,8]. Moreover, socio-economic factors have been identified as risk factors for SARS-CoV-2 infection [5,9].

This study took place during the first phase of the pandemic and before vaccinations became available. At that time most infections were caused by the wild type SARS-CoV-2 while the Alpha variant started spreading in Germany from December 2020 and became dominant within the following 3 months. It was performed in an unselected COVID-19-asymptomatic paediatric population in a Western German metropolitan region where ca 40% of families have migration background. The study had been set up to identify risk factors for SARS-CoV-2 infection in children and adolescents with the aim to identify populations at risk in Germany and to introduce preventive measures for the affected children and their families.

## Methods

### Study design and participants

In a standardised approach from 1 June 2020 to 1 February 2021, all asymptomatic children and adolescents from 6 months to 18 years of age who attended outpatient paediatric practices in three regions of Germany for scheduled mandatory routine examinations were invited to participate in the study (Corkid study, 'Corona in Kids') [3]. Participants and their parents were asked to fill in an electronic questionnaire (pre-installed on a tablet) with 14 questions (see Supplementary material) available in five different languages (German, English, Turkish, Arabic and Russian). It also asked about former SARS-CoV-2 infections (either symptomatic or asymptomatic). Recalled symptoms were categorised as, among others, fever, cough, loss of smell and/or taste, influenza-like illness and diarrhoea. Medical history as well as information on socio-economic and migration background (Germany/Western Europe, Eastern and Southern Europe, Turkey, Middle East, Commonwealth of Independent States (CIS states) America and Asia) of parents and grandparents was collected. Western European background included Austria, Belgium, France, Liechtenstein, Luxembourg, Monaco, the Netherlands and Switzerland; for this study, we did not classify individuals from these countries as ‘migrants’ because of comprehensive similarities in education system, standard of living and healthcare system. Southern Europe comprised Greece, Italy, Malta, Portugal and Spain. Eastern Europe included Albania, Bulgaria, Croatia, Hungary, North Macedonia, Poland, Romania, Slovakia and Slovenia.

Education level was defined as the highest level of education in terms of graduation of one of the parents/guardians. High level of education referred to high school/grammar school (Fachhochschule/Abitur), medium-level education to general secondary school (Realschule) and low-level education included education up to primary school/basic secondary school (Grundschule/Hauptschule). No educational degree (school education not completed, i.e. without final graduation) formed a fourth group. In addition, we collected information on the parent's working background and included high-risk working conditions with several hours of contact with many people per day (public transport, sales assistance, medical or nursing profession, teaching or childcare). We documented both the number of persons and the number of available rooms per household (rooms per person). History of chronic diseases and medication of the children was obtained from parents and verified by matching with medical charts. Accompanying parents were also invited to participate in the study, fill in the questionnaire and have their SARS-CoV-2 antibodies measured.

Blood samples were taken from all participating children on the occasion of their mandatory medical appointment. Serum samples were analysed for SARS-CoV-2 IgM and IgG nucleocapsid (N) antibodies (Elecsys Anti-SARS-CoV-2, Roche, sensitivity 99.5%, specificity 99.8%) using the manufacturer's cut-off. The target variable was SARS-CoV-2 infection, either given by seropositivity or by previous positive PCR test results reported by the family; however, PCR testing was not performed as part of the study. We aimed at comparing demographical data (age, sex), underlying medical conditions, socio-economic parameters and migration background between children and adolescents with and without evidence of SARS-CoV-2 infection.

### Statistical analysis

Demographic and clinical parameters between groups are presented with univariate odds ratios (OR) and 95% confidence intervals (CI). The impact of migration background was analysed by means of an adjusted random effects logistic regression. Thereby, we adjusted for the ratio of number of available rooms vs the number of persons living in the same household as well as for the highest educational level in the family. The ratio of rooms per person was introduced following the rationale that the available space per person is the essential surrogate for crowded living conditions and, therefore, a potential risk factor for transmission. Some families participated with more than one child in the Corkid study. Therefore, we included a random effect term in our model given by a family identification number. Insignificance of other factors was shown by a likelihood ratio-based model reduction process. Consequently, irrelevant factors were dropped in order to avoid overfitting. For the random effect analysis, we used the lme4 software package within statistical programming language R [10]. We considered p values under 0.05 as statistically significant.

## Results

### Demographics and clinical characteristics

In this cohort, 2,848 children and adolescents were asked initially to participate and 25% refused, mainly because of fear of drawing blood.

Of the remaining 2,124 (1,019 female; 48%) with a median age of 7.1 years (range: 0.6–18 years), 58 (2.7%) had evidence of SARS-CoV-2 infection (33 by SARS-CoV-2 antibody test only, six by PCR only and 19 by both methods). Our cohort exhibited an increasing seroprevalence rate from 0.5% in mid-2020 to almost 6% at the beginning of 2021 [3]. There were no clinical differences regarding sex, age or weight of positive cases compared with the SARS-CoV-2-negative children. Likewise, the number of children with pre-existing conditions (8/58 vs 381/2,066; 14 vs 18%) did not differ much between the groups. Twenty-four of the 58 infected children did not recall any infection during the preceding 3 months. Clinical characteristics of the participants by group are displayed in Table 1.

**Table 1:** Demographic and clinical data of study participants, SARS-CoV-2 infection in children, Germany, June 2020–February 2021 (n = 2,124)

	All		SARS-CoV-2-positive		SARS-CoV-2-negative		OR (SARS-CoV-2 yes/no)
	n	%	n	%	n	%	95% CI
Total	2,124		58		2,066		NA
Female	1,020	48.0	27	46.6	993	48.1	0.94 (0.56–1.59)
Male	1,104	52.0	31	53.4	1,073	52.9	0,92 (0,45–1,65)
≤ 3 years^a^	429	20.2	13	22.4	416	20.1	1.15 (0.61–2.14)
4–6 years (preschool)	817	38.5	22	37.9	795	38.5	0.98 (0.57–1.67)
7–10 years (primary school)	290	13.7	8	13.8	282	13.7	1.01 (0.47–2.16)
11–18 years (secondary school)	588	27.7	15	25.9	573	27.7	0.91 (0.5–1.65)
Underweight (< 3 percentile)	159	7.5	3	5.2	156	7.6	0.67 (0.21–2.16)
Obesity (> 97% percentile)	156	7.3	4	6.9	152	7.4	0.93 (0.33–2.61)
Any chronic medical condition^b^	389	18.3	8	13.8	381	18.4	0.71 (0.33–1.5)
Chronic airway disease	211	9.9	4	6.9	207	10.0	0.67 (0.24–1.86)

CI: confidence interval; NA: not applicable; OR: odds ratio; SARS-CoV-2: severe acute respiratory syndrome coronavirus 2.

^a^ In our cohort during the study period, only 71 children (16.5%) and only 1/13 of the children who tested positive for SARS-CoV-2 attended day care regularly.

^b^ All medical conditions requiring constant medical attendance and/or medication.

### Socio-economic and migration background

Because information on migration background was not documented for 51 participants, the following analysis is based on the remaining 2,073 patients. Within the remaining study cohort, 816 (39%) children and adolescents had a migration background (Table 2). While 4.4 (36/816) of children and adolescents with a migration background had evidence of SARS-CoV-2 infection, only 1.6% (20/1,257 of children with Western European background had evidence of infection (OR = 2.78; 95% CI: 1.63–4.74). Details about the socio-economic and migration background of the participants are shown in Table 2.

**Table 2:** Socio-economic parameters and migration background of study participants, SARS-CoV-2 infection in children, Germany, June 2020–February 2021 (n = 2,073)

	All			SARS-CoV-2-positive		SARS-CoV-2-negative	
	n	%		n	%	n	%
Total	2,073			56		2,017	
Parent SARS-CoV-2-positive^a^	59		3.0	27	50.0	32	1.7
SARS-CoV-2 contact^a^	268		14.5	33	64.7	235	13.1
Parents in high-risk occupations for SARS-CoV-2^a^	1,024		53.3	28	56.0	996	53.2
Parents' highest educational degree							
High school/grammar school	1,408		67.9	32	57.1	1,376	68.2
General secondary school	463		22.3	12	21.4	451	22.4
Basic secondary/primary school	132		6.4	9	16.1	123	6.1
No educational degree	70		3.4	3	5.4	67	3.3
Any migration background in any of parents/grandparents	816		39.4	36	64.3	780	38.7
Turkey	180		8.7	13	23.2	167	8.3
Middle East	108		5.2	8	14.3	100	5.0
Asia	28		1.4	3	5.4	25	1.2
Commonwealth of Independent States (CIS states)	127		6.1	3	5.4	124	6.1
Eastern Europe	288		13.8	7	12.5	281	13.9
Other	85		4.1	2	3.6	83	4.1
Germany/Western Europe	1,257		60.6	20	35.7	1,237	61.3
Members in the household^a^							
1–3	564		27.2	13	23.2	551	27.5
4–5	1,337		64.8	36	64.3	1,301	64.8
> 5	162		7.9	7	12.5	155	7.7
Rooms in the household^a^							
1–3	522		25.4	24	42.9	498	24.9
4–5	1,029		50.0	23	41.1	1,006	50.3
> 5	505		24.6	9	16.1	496	24.8

SARS-CoV-2: severe acute respiratory syndrome coronavirus 2.

^a^ Missing value. Denominator for the percentages excludes study participants with missing information.

A random effect logistic regression yielded the results listed in Table 3. Due to missing records for the numbers of rooms in the household and for family members, the regression was based on 2,052 instead of 2,073 observations. Specifically, the highest risks for infection were detected in children of Turkish (OR = 6.24; 95% CI: 1.38–28.13; p = 0.017) and Middle Eastern (OR = 6.44; 95% CI: 1.14–36.45; p = 0.035) origin. In the Asian population, seroprevalence was also high (OR = 16.6; 95% CI: 1.07–250), but because only 1.4% of the cohort originated from this region this result must be interpreted with caution.

**Table 3:** Random effect logistic regression and univariate analysis, SARS-CoV-2 infection in children, Germany, June 2020–February 2021 (n = 2,052)

Predictors	OR (univariate)	p value	OR (logistic regression)	95% CI	p value
Background					
Germany/Western Europe	Reference		1.0	Reference	
Turkey	4.81	< 0.001	6.24	1.38–28.13	0.017
Middle East	4.95	< 0.001	6.44	1.14- 36.45	0.035
Eastern Europe	1.54	> 0,05	1.56	0.42–5.78	0.503
Asia	7.42	0.002	16.36	1.07–250.36	0.045
Living conditions					
Rooms per person	0.25	0.001	0.35	0.08–1.5	0.158
Level of education					
High level	Reference		1.0	Reference	
Medium level	1.08	> 0,05	1.02	0.37–2.81	0.972
Low level	3.15	0.003	3.13	0.72–13.69	0.129
No educational degree	1.6	> 0,05	1.63	0.23–11.69	0.628

CI: confidence interval; OR: odds ratio; SARS-CoV-2: severe acute respiratory syndrome coronavirus 2.

Other risk factors relevant for risk adjustment, although below significance, included low level education of the parents (OR = 3.13; 95% CI: 0.72–13.69) and crowded living conditions, inversely expressed by rooms per person (OR = 0.35; 95% CI: 0.72–13.69). Of note, these two factors had statistically significant effects in univariate analyses (Table 3).

## Discussion

This population-based study identified seropositivity rates and risk factors for SARS-CoV-2 infections in asymptomatic children and adolescents who attended legally required preventive examinations. Our key findings were: (i) Less than half of the seropositive participants of all age groups recalled symptoms of infection within the 3 months preceding the study, suggesting that more than half of SARS-CoV-2 infections go unnoticed [3]. (ii) The cumulative incidence reflected by prevalence of antibodies in serum of SARS-CoV-2 infections was significantly higher in families of Turkish or Middle Eastern descent irrespective of other risk factors, i.e. after adjustment for other socio-economic confounders.

As in other seroprevalence data from German or Swiss cohorts, we observed an increase in seroprevalence from 0.5 to 6% during the study period [2,11,12]. The dynamics of this increase in the seroprevalence of SARS-CoV-2 infections match well with national data of acute SARS-CoV-2 infection in the same geographical region [4,13]. Infection rates were comparable across all age groups in keeping with the findings of other studies [1,4,8].

It is not surprising that known exposure to SARS-CoV-2-positive individuals doubled the risk for seroconversion. In most families, at least one parent also had evidence of SARS-CoV-2 infection, which is slightly more than described in other cohorts [14,15]. However, half of these SARS-CoV-2-exposed children did not develop evidence of infection (seronegative).

Recent studies from the US state that socio-economically disadvantaged children, especially those from ethnic minorities, are at higher risk of infection [5,7,8]. Data from the United Kingdom (UK) and the US also show increased morbidity and mortality from SARS-CoV-2 in adults from ethnic minorities and with poor socio-economic status [6,9]. Limited access to healthcare systems and migration background might also play a role in spreading the disease [16,17].

The most relevant risk factor for SARS-CoV-2 infection in our study population was a Turkish or Middle Eastern migration background. Although socio-economic factors confound the factor of migration history to some extent, our findings result from an adjusted regression model and are therefore independent of educational standard and housing conditions. Reasons for this observation could include different family and social structures favouring closer contacts and therefore increasing the risk of transmission as described by Gaskell et al. for a tight Jewish Orthodox community in the US [18]. Apart from that, language as well as cultural barriers might play a role [9] as well as travelling habits. Visiting friends and relatives in countries with higher incidence of infection might further increase the risk. Scepticism regarding politics and health authorities are an additional issue, especially in prevention and vaccination programmes [1]. To approach these families, tailored, culture-sensitive strategies need to be developed.

In keeping with data from other studies on seropositivity in children and families [1,2,11,12] and with observations in other populations with a migration background e.g. in the UK or the US [5-8], we here provide data from a region of Germany with a high percentage of families with a migration background. We assume that our results can help identify opportunities for timely targeted interventions.

Our study had limitations: Paediatric patients were recruited only from three regions in Germany, and not all paediatric practices in the area participated. In addition, initially low seroprevalence rates could have introduced a bias. The proportion of children from Turkish families, in contrast to those of other origins, was 9%, lower than expected seeing as 20% of the families in the regions studied are of Turkish origin [19]. However, this rather indicates an underestimation of the true infection rate in this group. The migration status was not differentiated with regards to legal status or number of years lived in Germany. In some of the calculations regarding infection risk in children with migration background, 95% CI were wide and the findings have to be interpreted with caution. Another limitation might be the use of surrogate parameters for the assessment of living conditions and income, such as the number of rooms etc. Different educational backgrounds not matching the German system (participants who had part of their education in other countries) might have posed difficulties in answering questions about educational status and might have also led to an underestimation of the corresponding risk factor. We attempted to reduce a possible bias through semi structured interviews to validate the answers drawn from the Internet-based questionnaire. In addition to this, we included children and adolescents from a wide range of ages (0–18 years). Different mechanism of transmission may have played a role in different age groups.

## Conclusions

This study shows that Turkish or Middle Eastern migration background even after correcting for poor socio-economic status, were risk factors for SARS-CoV-2 infection in a population-based cohort of children and adolescents in Germany. Targeted interventions and tailored prevention strategies in these groups are necessary to improve infection control and to protect these vulnerable populations from excess morbidity and mortality. They could serve as a blueprint for vaccination programmes, and there is a need to shift the focus of politics towards more culture-sensitive interventions.

## Supplementary Data

205000 Supplement
