# Supplementary material for: Seroconversion rate and socio-economic and ethnic risk factors for SARS-CoV-2 infection in children in a population-based cohort, Germany, June 2020 to February 2021
Source: Euro Surveill. 2022 Sep 15;27(37):2101028. doi: 10.2807/1560-7917.ES.2022.27.37.2101028 (PMC9479468; doi:10.2807/1560-7917.ES.2022.27.37.2101028)
Supplement: Supplement [file 2101028_BRINKMANN_Supplementary_material.pdf]

Supplement 1

This supplementary material is hosted by Eurosurveillance as supporting information alongside the article “Seroconversion rate and socio-economic and ethnic risk factors for SARS-CoV-2 infection in children in a population-based cohort, Germany, June 2020 to February 2021”, on behalf of the authors, who remain responsible for the accuracy and appropriateness of the content. The same standards for ethics, copyright, attributions and permissions as for the article apply. Supplements are not edited by Eurosurveillance and the journal is not responsible for the maintenance of any links or email addresses provided therein

CorKid

Serokonversionrate von SARS-CoV-2 bei Kindern und Jugendlichen und ihren Eltern im Ruhrgebiet

Fragebogen zu Beginn (Tablett)

ID : wird vergeben

Datum : Praxis: Default

(von Arzthelferin vorausgefüllt)

Geplante U- Untersuchung: Liste U4, U5, U6, U7, U8, U9 , J1, J2

Geschlecht: w/m/d

Liebe Eltern, wie angekündigt erhalten Sie jetzt einen Fragebogen. Wir würden uns freuen, wenn Sie ihn jetzt schon ausfüllen oder, wenn Sie wollen, erst nach der Untersuchung Ihres Kindes. Wenn nach der Untersuchung die Teilnahme ablehnen, werden allen personenbezogen Angaben, die in den Kästen stehen, direkt wieder gelöscht. Wir behalten diese Angaben bei einer Teilnahme auch nur so lange, bis die Studie beendet ist und wir Ihnen alle Ergebnisse zurückmelden konnten (siehe Aufklärungsbogen), danach werden sie ebenfalls gelöscht

Kind: Name: Vorname:

Geburtsdatum:

Begleitperson : Mutter Vater sonstige

Name: Vorname:

Telefonnummer (Festnetz) : Telefonnummer (Handy):

Um wieviel Uhr meistens erreichbar : egal

Bevorzugt morgen/mittags/nachmittags/ab 18 Uhr /20-21 Uhr

Emailadresse : …………….@..................

1. Wie viele Kinder haben Sie?

2. Wie viele Personen leben bei Ihnen im Haushalt?

3. Wurde bei Ihrem Kind ein Abstrich wegen Verdacht auf Covid19 Infektion gemacht?
   Nein □ ja □ weiß ich nicht □
   Wenn ja
   3.1. wann : (Monat) / 2020
   3.2. Wie war das Ergebnis: *negativ /positiv*
   3.3. Wurde der Test wiederholt? *Nein* □ *ja* □ weiß ich nicht □
   Wenn ja
   3.3.1. Wie oft
   3.4. Wie war das Ergebnis: *negativ /positiv*

4. Während der Zeit der Kontaktsperre war Ihr Kind
   überwiegend zu Hause? □
   in der Notbetreuung? □
   sonstiges? □

5. Hatten Sie oder andere enge Familienmitglieder im gleichen Haushalt seit 02/2020 Kontakt zu einem COVID- 19 Infizierten oder Erkrankten?
   Nein □ ja □

6. Hatte Ihr Kind einen oder mehrere Infekte in den letzten 3 Monaten?

   Nein □ ja □ weiß ich nicht □

   Wenn ja:

   6.1. Wie viele Infekte: *einer □ zwei □*drei □ *mehr oder dauerhaft □*
   6.2. Hatte Ihr Kind bei einem der Infekte eine oder mehrere der folgender Beschwerden (Symptome). (Sie können mehrere Kästchen ankreuzen)

   (die folgenden Fragen wiederholen für Infekt 1 bis 3, je nach Antwort auf die Frage 6.1)

   6.2.1. Fieber > 38,5°C Nein □ ja □… *weiß ich nicht □*…
   6.2.1.1. Wenn ja wie viele Tage Fieber…………
   6.2.2. Schnupfen Nein □ ja □… *weiß ich nicht □*.
   6.2.3. Halsschmerzen Nein □ ja □… *weiß ich nicht □*.
   6.2.4. Husten Nein □ ja □ …. *weiß ich nicht □*
   6.2.5. Atemnot Nein □ ja □…. *weiß ich nicht □*
   6.2.6. Kopfschmerzen Nein □ ja □… *weiß ich nicht □*
   6.2.7. Müdigkeit/ Abgeschlagenheit Nein □ ja □ . *weiß ich nicht □*.
   6.2.8. Durchfall/Erbrechen Nein □ ja □ *weiß ich nicht □*…
   6.2.9. Geschmacks/Geruchsstörung Nein □ ja □… *weiß ich nicht □*
   6.2.10. Dauer der Beschwerden (Tage)…………….

6.2.11. Wieviel Tage im Krankenhaus gar nicht wie viele Tage ……….

7. Ist ein Familienmitglied in einem Bereich mit viel Menschenkontakt tätig, (Altenpflege, Krankenhaus, Supermarkt, Taxi,..) nein ja

8. Hat Ihr Kind eine dauerhafte Erkrankung Nein ja … *weiß ich nicht*
   Wenn ja:
   Welche ? : ……………………………………… *(Freitext)*

9. Braucht Ihr Kind derzeit regelmäßig Medikamente Nein ja … *weiß ich nicht*
   Wenn ja
   9.1. Welche ? : ……………………………………… *(Freitext) (mehrere Zeilen)*

Vielen Dank

Wenn Sie mit der Kinderärztin oder Kinderarzt gesprochen haben, würden wir uns freuen ,wenn Sie an der CorKID Studie teilnehmen. Dafür erhalten Sie dann eine Einverständniserklärung. Wenn Sie nicht teilnehmen wollen, werden alle personen-bezogen Angaben, die oben in einem Kasten stehen, gelöscht.

<u>Fragen an die Kinderärztin, den Kinderarzt (wir ausgedruckt von der Helferin eingeben):</u>

10. Gab es auffällige /pathologische Befunde bei der U-Untersuchung ? Nein ja
    10.1. Wenn ja: welche……………………………………………….(Freitext)
11. Sind chronische Krankheit bekannt Nein ja
    11.1. Wenn ja: welche……………………………………………….(Freitext)
    11.2. Aktuelle Dauermedikation …………………….. (Freitext, mehrere Zeilen)
12. Eltern der Teilnahme zugestimmt ? Nein ja (Extra-Ausdruck mit Unterschrift Eltern(Kind/KÄ KA
13. Impfungen:
    Vollständig (lt. STIKO)
    Unvollständig
    Influenza Impfung 2019/2020
14. Bemerkungen………… (Freitext)
